# Carcass use by mesoscavengers varied across modified landscapes in the absence of top carnivores

**DOI:** 10.1007/s00442-025-05697-1

**Published:** 2025-04-03

**Authors:** Matthew W. Fielding, Luke A. Yates, Jessie C. Buettel, Dejan Stojanovic, Barry W. Brook

**Affiliations:** 1 School of Natural Sciences, University of Tasmania, TAS, Private Bag 5, Sandy Bay, 7001 Australia; 2 ARC Centre of Excellence for Australian Biodiversity and Heritage, Wollongong, Australia; 3 Fenner School of Environment and Society, Australian National University, Canberra, Australia

**Keywords:** Scavenging, Land-use change, Feral cats, Carcass use, Carrion

## Abstract

The decomposition of carrion is crucial to the functioning and nutrient cycling of ecosystems, and many species use this high-quality resource. However, the availability and reliability of carrion differs across environments. Modified landscapes, such as farms and roads, often produce a high density of carcasses, with disproportionate benefits for generalist mesoscavengers that can tolerate, or are attracted to, human presence. In this study, we placed carcasses strategically across two large islands in southern Australia and used camera traps to investigate how different scavengers locate and use carrion in forested, farmland and roadside habitats. Forest ravens, an avian generalist, were the dominant scavengers across all three habitat types but were three times more likely to discover carcasses deployed in modified landscapes. Raptors were twice as likely to discover and use carcasses in farmland habitats, indicating enhanced scavenging opportunities in these areas. The discovery rate and use of carcasses by feral cats did not differ between habitat types. In this setting, differences in carrion use between diurnal and nocturnal scavengers are potentially due to the absence of top mammalian carnivores (Tasmanian devils and quolls). Diurnal scavengers, such as forest ravens and raptors, followed expected habitat preferences, albeit with greater access to carrion due to increased persistence. However, feral cats demonstrated no preference for carcasses across habitats, likely due to the reduced landscape of fear. Overall, our study underscores the important role of native mammalian carnivores in structuring scavenger communities and further validates the value of trophic rewilding to restore key ecological functions.

**Supplementary Information:**

The online version contains supplementary material available at 10.1007/s00442-025-05697-1.

## Introduction

Carrion is a vital resource for many species across the world (Barton et al. 2013). Not only does it provide food for scavengers, invertebrates, and microbes, but the decomposition of carrion is also important for the structuring of an ecosystem and its communities (Ripple et al. 2014; Wilson and Wolkovich 2011). As carrion is nutritionally rich, there is often intense competition for the resource between scavengers (O'Bryan et al. 2019). Carrion can be used as an indicator of ecosystem health by providing insights into the functioning of the food web (Newsome et al. 2021). Obligate scavengers—those species that only scavenge, like vultures—play an important role in regulating the access of carrion to other species (Buechley and Şekercioğlu 2016). In contrast, many apex predators are facultative (opportunistic) scavengers, but their presence within an ecosystem is nevertheless vital for regulating access to carcasses and thus ‘trophic limitation’ (Allen et al. 2014). However, with populations of larger scavengers declining globally because of habitat loss and human persecution, mesoscavengers are increasing in abundance due to a reprieve from competition (Buechley and Şekercioğlu 2016; Kuijper et al. 2016; O'Bryan et al. 2019). In addition, modified landscapes often offer more reliable scavenging opportunities, for example through culling programmes or roadkill, potentially providing further benefits to generalist mesoscavengers (Newsome et al. 2015; Read and Wilson 2004; Sebastián-González et al. 2019).

Land-use change has occurred in many natural ecosystems, for example via vegetation clearance to create urban and agricultural areas (Winkler et al. 2021). This often has negative impacts on species, removing vital habitat and excluding them from specific areas (Hepinstall et al. 2008; Marzluff et al. 2001; McKinney 2006). In addition, clearing is facilitated by extensive road networks which continue to grow across the world (Ibisch et al. 2016; Laurance et al. 2014). These road networks can impact species by creating a fragmented mosaic of modified-natural land, but can also directly lead to the death of species from vehicle collisions, with hundreds of millions of animals killed on roads each year (Benitez-Lopez et al. 2010; Fahrig and Rytwinski 2009; Rytwinski et al. 2016). Culling in agricultural areas to deal with overabundant herbivores, paired with increased vehicle–animal collisions, has led to a surplus of reliable carrion around farms (Forman and Alexander 1998; Morelli et al. 2014; Schwartz et al. 2018; Welti et al. 2019) with many scavengers appearing to benefit from abundant food (Lambertucci et al. 2009; Peisley et al. 2017; Planillo et al. 2015). In addition, the competitive balance for carrion appears to differ in these modified landscapes, with mesoscavengers rather than large-bodied specialists being the predominant scavengers (Devault et al. 2011). However, the rates of discovery and use of carcasses by mesoscavengers between roads, farms and forested habitats, without any top-down control of top carnivores have yet to be quantified.

Following European occupation of the Bass Strait region (between the Australian mainland and Tasmania) the largest islands, King Island (1,098 km^2^) and Flinders Island (1,367 km^2^), have been extensively modified. For example, over two-thirds of King Island’s pre-European native vegetation has been cleared for agriculture (Threatened Species Section 2012). This has led to several local extinctions, including the loss of all native mammalian carnivores, such as the spotted-tailed quoll *Dasyurus maculatus* (Fielding et al. 2020; Peacock et al. 2018). The absence of predation by larger mammalian predators, along with abundant food from artificial pastures, has caused a concomitant population boom in herbivores with an estimated half a million macropods on the islands (Branson 2008; Norton and Johannsohn 2010). This has led to increases in roadkill, as well as carcasses of wallabies that are culled in paddocks to control numbers, and thereafter left to decay where they fall (Fielding et al. 2021a; Threatened Species Section 2012). Therefore, there is an abundance of carrion for the remaining scavengers, such as the invasive feral cat (*Felis catus*) and native facultative avian scavengers, such as raptors and the forest raven (*Corvus tasmanicus*), which could lead to augmentation of their populations (Fielding et al. 2022). As known predators of small birds, an increased abundance of feral cats and forest ravens could lead to further attacks and pressure on several endangered avian species found on the islands (Debus and Rose 2006; Dickman 1996; Ekanayake et al. 2015; Talmage 2011; Webb et al. 2016). Forest ravens are also known to attack livestock and raid crop plants, such as vineyards, particularly when present in higher densities (Rowley 1969; Rowley and Vestjens 1973). This atypical setting, without any apex mammalian scavengers, provides a rare opportunity to understand how smaller scavengers use carcasses across habitats, within the context of a simple scavenger community structure, and offers insights into the impacts of the loss of larger-bodied mammalian carnivores on mesoscavengers.

In this study, we deployed carcasses and camera traps using a blocking design to monitor carrion use by scavengers across three habitat types, forests, farmland, and roadsides. Using these methods, we tested the following predictions: (1) carrion discovery by invasive and avian mesoscavengers will differ across habitat types, with carcasses in modified landscapes (with greater visibility) being found earlier; (2) carcass use and scavenging time by invasive and avian mesoscavengers will be greater in modified landscapes compared to natural environments because of increased accessibility; (3) carcasses will persist within natural environments for longer due to reduced scavenging by mesoscavengers because of factors (1) and (2); and (4) forest ravens will be the dominant scavenger across all land use types, due to the absence of native mammalian carnivores and because of their elevated abundance across the landscape.

## Methods

### Study area and experimental design

**Fig. 1:**
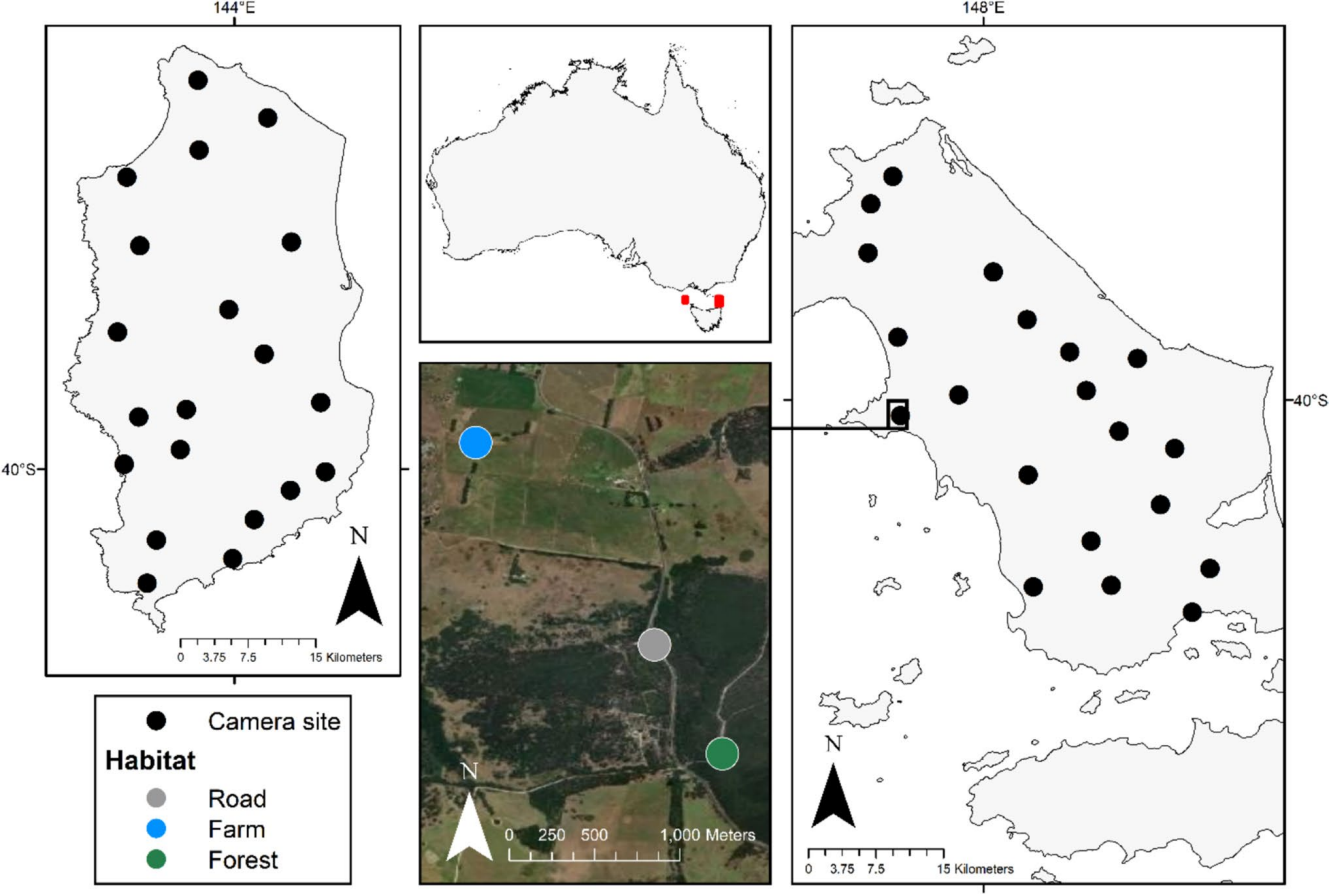
Geographic location of study sites across the Bass Strait Islands. The red indicators show the locations of King Island (left) and Flinders Island (right). Each black circle corresponds to a block. Within each block, we stratified the three habitat types (farm, road and forest) and placed a camera and a carcass at each site. The satellite image shows an example of a block, where three different sites of different habitats were selected in close proximity to one another

We measured scavenger use of carrion in three habitat types across the Bass Strait Islands in south-eastern Australia (Fig. 1). The Bass Strait Islands have repeatedly been part of a land bridge connecting Tasmania and the Australian mainland, most recently during the last glacial maximum until around 12,000 years ago (Adeleye et al. 2021; Hope 1973). Consequently, they share fauna and flora with the mainland; however, the extirpation of quolls and Tasmanian devils (*Sarcophilus harrisii*) on the Bass Strait Islands means that native mammalian predators are absent (Peacock et al. 2018). Since European occupation in the 1800s, the islands have been heavily modified for agriculture, leading to large expanses of pasture and an extensive road network (Threatened Species Section 2012). We used the mosaic of modified and natural land on King Island and Flinders Island to identify three different habitats: (i) natural forest/scrub; (ii) modified open pasture; (iii) roadside, adjacent to either natural forest, open pastures, or both (Fig. 1). We used a blocking design to stratify our study area and control for spatial autocorrelation by creating a relatively even distribution of points across both islands. Within each block, we selected three nearby sites corresponding to forest, farm, and roadside habitats. Within each habitat, we deployed a camera and a carcass (see below for details). In total, we had 40 blocks, and thus we had 40 replicates of each habitat type (comprising a sample of 120 cameras). Blocks were separated by at least 2 km, but sites within a block were always within 2 km of one another.

The carcasses and camera traps were deployed during August—September 2020. Late winter was chosen because that is when invertebrate and microbial decomposition is lowest. At each camera, we placed an individual Bennett’s wallaby (*Macropus rufogriseus*; 13.8–18.6 kg) or Tasmanian pademelon (*Thylogale billardierii*; 1.5–8 kg) carcass. These species are regularly culled on the islands under crop protection permits, and we sourced freshly culled animals from licenced shooters. We used Cuddeback X-Change 1279 camera traps, which were deployed for a minimum of 21 days, after which we expected the carcasses to be mostly consumed based on findings from similar studies (Cunningham et al. 2018; Newsome and Spencer 2022). Due to similarities in behaviour and functional roles (Marchant and Higgins 1993), we grouped all observed raptor species (listed in order of numbers of observations: white-bellied sea eagle *Haliaeetus leucogaster [n* = 23*]*, swamp harrier *Circus approximans* [*n* = 20]*,* wedge-tailed eagle *Aquila audax [n* = 16*]*, and brown falcon *Falco berigora [n* = 2*]*) for the analysis.

### Analysis

#### Carcass discovery and persistence

We used R v4.0.2 for all analyses (R Core Team 2020). We defined discovery as the first instance in which an animal found and scavenged on the carcass. A carcass was classed as fully consumed when there was a clear final consumption event and less than ~ 5% of the carcass remained. To analyse how carcass discovery and persistence differed across habitat types, we used survival analysis with a semiparametric Cox proportional hazards regression model, implemented using the R package ‘survival’ (Therneau 2021). Survival analysis is useful when time-since- or time-until-event data are censored (Hosmer et al. 2008). Carcass discovery data were right-censored because not every carcass was discovered by each species before the end of the study period. In addition, several carcasses were removed by animals early from the camera field of view (*n* = 32), two cameras were knocked over before the carcass was consumed and the batteries in one camera died before the end of the study. We expected different patterns in the discovery and use of carcasses between species. Therefore, separate analyses were used to investigate the time it took for the carcasses to be discovered by (i) all vertebrate scavengers, (ii) ravens, (iii) all raptor species, and (iv) feral cats. We also analysed the persistence of each carcass within the environment.

For all analyses, we developed a suite of ecologically informed models (see Supplementary Information Table S1) containing combinations of a set of predictor variables including habitat (factor: forest, farmland, roadside) and several variables related to human-modified landscapes: 2020 human population density (extracted from Australian population grid 2020 from the Australian Bureau of Statistics: https://www.abs.gov.au/); distance to freshwater (km; extracted from Hydrology datasets from www.theLIST.tas.gov.au* ©State of Tasmania*) and distance to the coastline (km; calculated using digital boundaries from the Australian Bureau of Statistics: https://www.abs.gov.au/). For the analysis, we transformed the habitat multi-level factor into two binary dummy variables of road and farmland, meaning forest was represented when both variables were equal to 0. We included island as a categorical predictor in all models to control for unmeasured island-specific effects. For model comparison, we used leave-one-out cross-validation (LOOCV) to calculate estimates of the Kullback–Leibler discrepancy (Roberts et al. 2017). However, traditional LOOCV methods cannot be used for time-to-event data as the data require at least two observations in the test data set (Dai and Breheny 2019). To address this, we used an alternative LOOCV method for survival analysis developed by Verweij and Van Houwelingen (1993), in which the log partial likelihood for the training data is taken from the log partial likelihood of the entire data set. To avoid overfitting (due largely to model-selection uncertainty), we used a modified one-standard-error rule to select the most parsimonious model with predictive performance comparable to the best predictive model (Yates et al. 2021). To assess the variable effect size of covariates within each selected model, we calculated hazard ratios (HR; exponentiated coefficients) using the full model to mitigate selection-induced bias. For each model, we only reported the hazard ratio for island, as the control variable, if the variable had 95% confidence intervals that did not cross 1. For visualisation, carcass data were separated into the three habitat types and we used the packages ‘survival’ (Therneau 2021) and ‘survminer’ (Kassambara et al. 2021) to also compute a non-parametric Kaplan–Meier estimate of the survival function.

#### Carcass consumption and scavenging time

To analyse carcass consumption, we defined a binary variable representing whether or not a carcass was actively fed upon by a target species. For scavenging time, we defined a continuous variable of the total duration (minutes) a species spent actively feeding upon a carcass. To visualise the variation in both variables for each species between habitat types, we used the average proportion of carcasses used and average scavenging time within each habitat type. The bootstrap 95% confidence intervals were calculated by resampling (*n* = 10^5^) the observations for each habitat type (Efron and Tibshirani 1993). We removed 20 cameras from this component of the analysis because their data were incomplete.

To determine the predictors of carcass consumption by forest ravens, feral cats, and raptors we used Generalised Linear Models (GLMs). We tested a range of a priori models based on ecological knowledge (Table S1) which included the same predictor variables as the carcass-discovery analyses. Again, we accounted for any between-island variation by including it as a categorical predictor in all models. We modelled carcass discovery and subsequent carcass consumption as a two-step hurdle process (Feng 2021). The first step comprises a binomial GLM to model the binary response of carcass discovery. The second step, conditional on discovery, models total foraging time using a gamma-distributed GLM. We were unable to analyse the total foraging time for raptors and feral cats due to insufficient data, however, we used GLMs with a binomial link function to analyse carcass use by these species. To select the most parsimonious model, we again used LOOCV with the modified one-standard-error rule (Yates et al. 2021). We calculated the effect size (ES; predicted probability when the variable was applied against probability when the effect was absent) for all variables included in the best model. Again, we used estimates from the full model to minimise selection bias. For each analysis, we only reported the effect size of the control variable, island, when the values were more than two standard errors away from zero.

## Results

### Carcass discovery and persistence

**Fig. 2:**
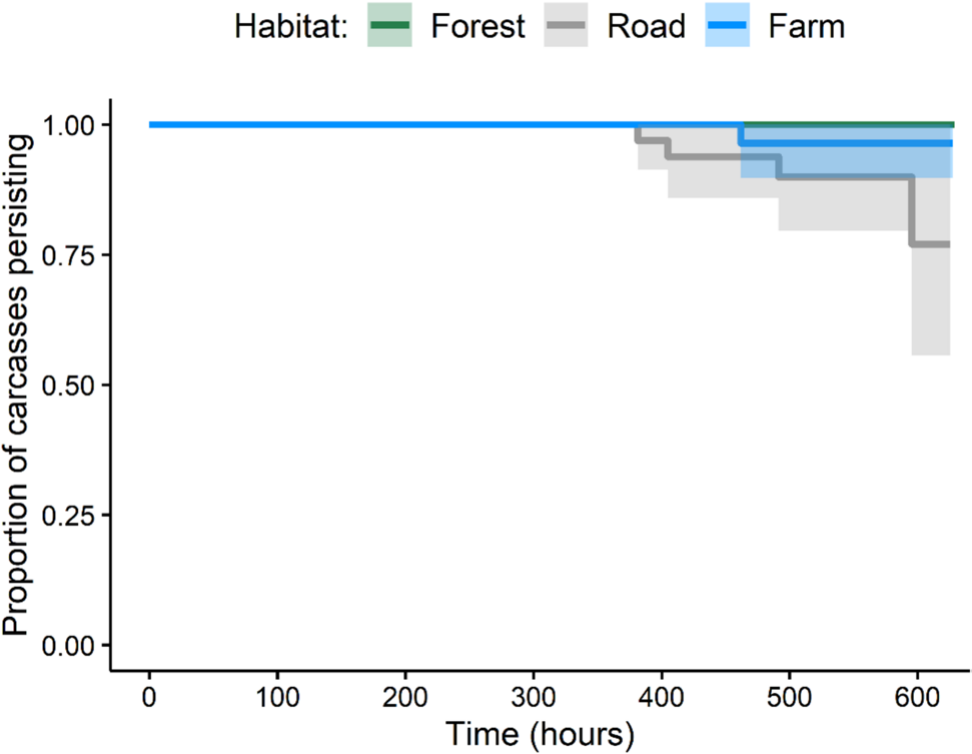
Time series of the proportion of carcasses persisting in the environment across habitat types. Colour shading indicates the 95% confidence interval

**Fig. 3:**
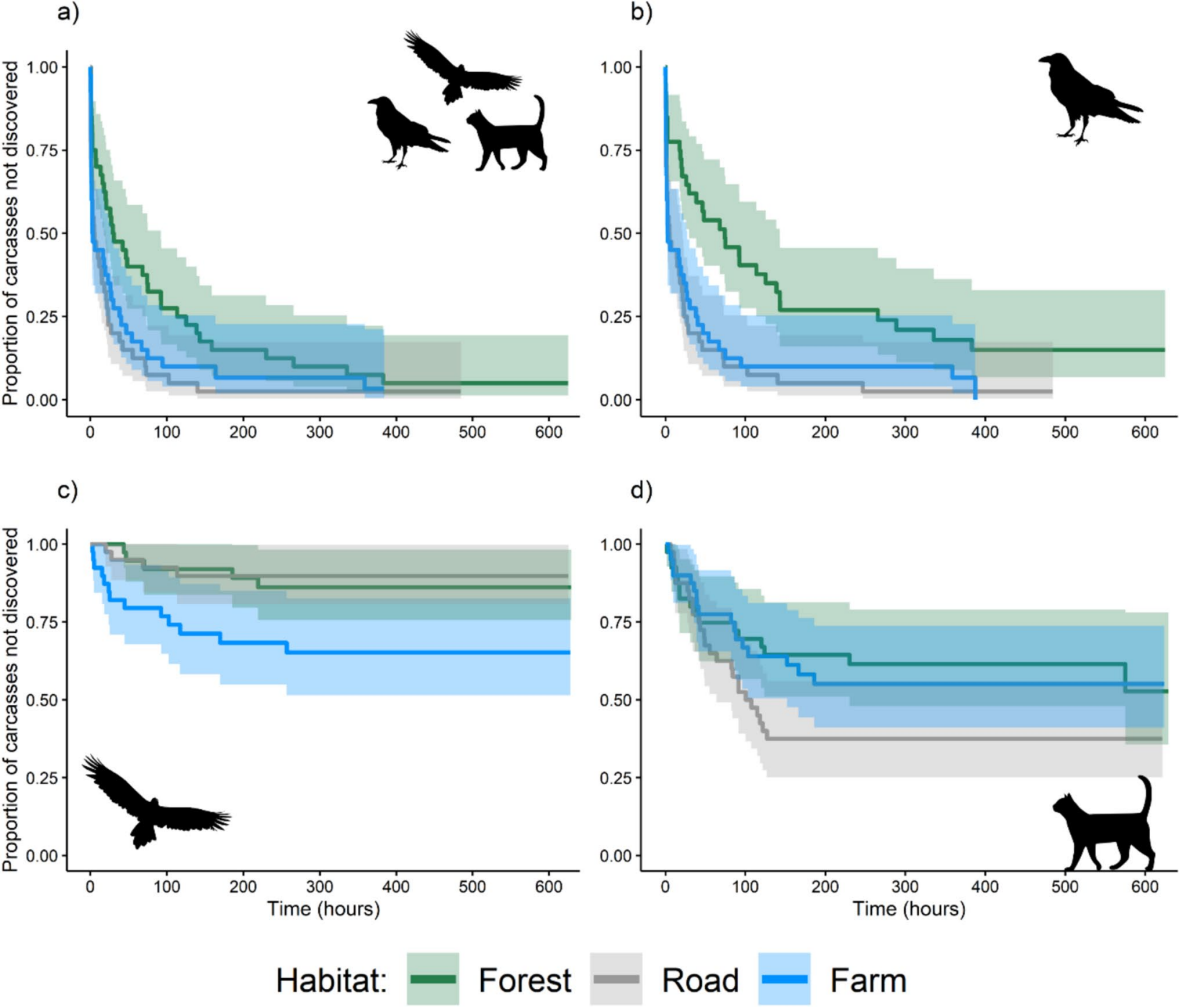
Kaplan–Meir estimates of the survival function for the proportion of carcasses discovered by **a** all species, **b** forest ravens, **c** raptors, and **d** feral cats across the three habitats. The 95% confidence interval is indicated by the shading

The persistence of carcasses across the three habitat types was homogeneous (Fig. 2) and the null model was preferred (see Table S2 and Fig. S1 for model selection results and Table S3 for model output). When all species were collated, there was no difference in the discovery of carcasses between the three habitat types (Fig. 3a). Ravens were more likely to discover carcasses on roads (HR = 2.54; 95% CI: 1.56–4.13) or farmland (HR = 2.20; 95% CI: 1.35–3.58) than forested areas (Fig. 3b). Raptors were more likely to find carcasses within farmland habitat (HR = 3.05; 95% CI: 1.06–8.80) compared to forested areas (Fig. 3c). Carcass discovery by feral cats did not differ between habitat types (Fig. 3d) and the null model was preferred.

### Carcass consumption and scavenging time

**Fig. 4:**
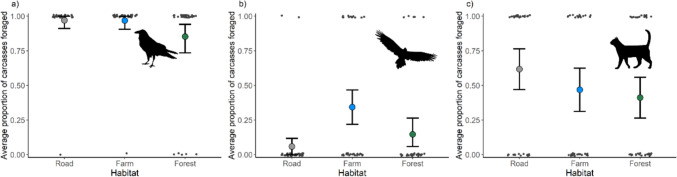
Carcass consumption by focal species across the three habitat types. The proportion of total carcasses foraged by **a** forest ravens, **b** all raptors and **c** feral cats. The value for each site is indicated by the black dots and the error bars are bootstrapped 95% confidence intervals

**Fig. 5:**
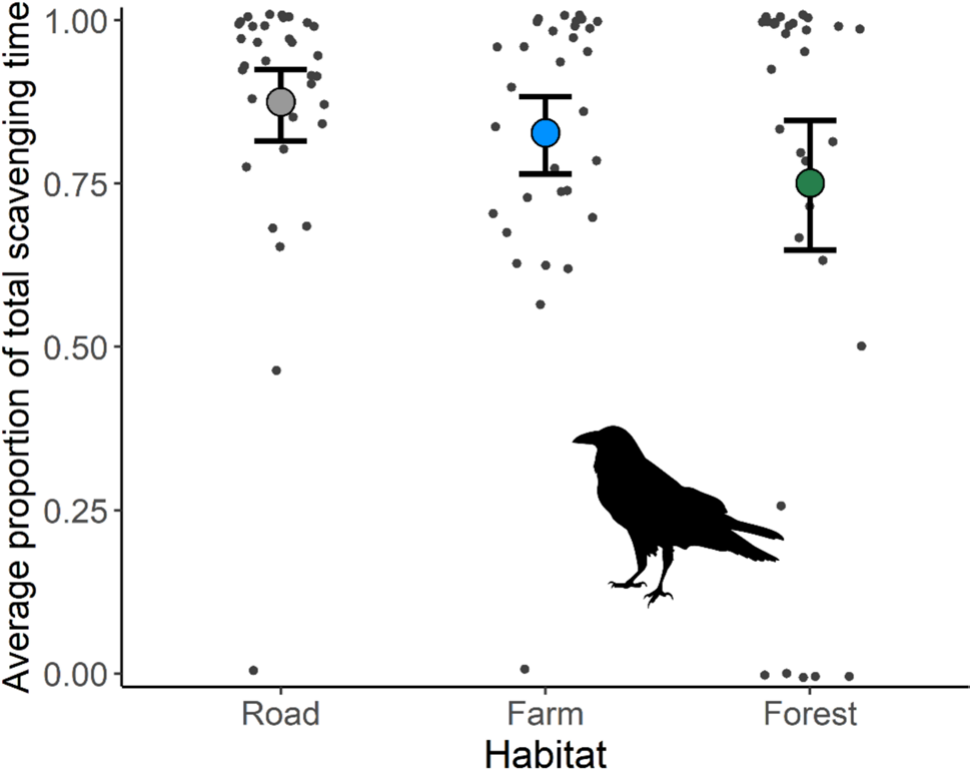
The average proportion of total scavenging time by forest ravens across the three habitat types. The values for each site are indicated by the black dots and the error bars are bootstrapped 95% confidence intervals

All species scavenged more carcasses in the modified landscapes (roadside and farmland) compared to the forested sites. Forest ravens were the dominant scavengers across all three habitat types. They found and scavenged on almost all the carcasses found on roadsides (*n* = 39; 97.5%) and farmland (*n* = 38; 95%), but only scavenged on 80% (*n* = 32) of forest carcasses (Fig. 4a). Further, forest ravens were the dominant scavenger across all three habitat types in the proportion of total time spent scavenging (roadside: 90.8%; farmland: 87.7%; forest: 88.3; Fig. 5). Interestingly, there were no supported predictors of carcass use by forest ravens, with the null model selected (see Table S4 and Fig. S2 for model selection results and Table S5 for model output). Similarly, there was no difference in scavenging time by forest ravens across habitat types. Raptors scavenged on 32.5% of farmland carcasses (*n* = 13), which was 3.25 times more than those on roadsides (*n* = 4; 10%) and 2.6 times more than those in forests (*n* = 5; 12.5%; Fig. 4b). Habitat influenced carcass use by raptors, with the group more likely to feed on carcasses in farmland habitats (ES: 2.85) than in roadside or forested areas. Further, raptors were more likely to use carcasses on Flinders Island (ES: 2.93) than on King Island. Feral cats fed on 62.5% (*n* = 25) of roadside carcasses, which was 1.5 times as many as those on farmland (*n* = 17; 42.5%) and 1.6 times as many as forest carcasses (*n* = 16; 40%). Despite this, there was no difference in carcass use by cats between habitats (Fig. 4c). However, feral cats were more likely to use carcasses on Flinders Island than on King Island (ES: 1.97).

## Discussion

We used strategically placed carcasses and camera traps to quantify carcass discovery and use by scavengers across modified and natural landscapes in an environment without large native mammalian scavengers. We revealed that small avian scavengers, ravens, used more carcasses on both farmland and roads, whereas large avian scavengers, raptors, discovered and used more carcasses within modified farmland landscapes. Whilst the invasive feral cat fed on more carcasses in modified landscapes, there was no difference in carcass discovery between habitat types. Despite this increased use of carcasses by scavengers in modified landscapes, almost all carcasses persisted until the end of the study period. This could be related to the lack of any native mammalian carnivores within our study system, potentially leading to increases in carrion-borne diseases where these species are absent. These results highlight the disproportionate benefits that native mammalian carnivore loss seems to present to mesoscavengers, such as ravens and feral cats. These findings provide support for carrion management strategies, such as carcass removal, and the reintroduction of top native carnivores.

Forest ravens were the dominant scavengers across all three habitat types (Figs. 3b, 4a, 5). Extensive land clearing has been beneficial for ravens, as they readily adapt to new environments and exploit new resources easily (Rowley and Vestjens 1973). Like most avian scavengers, ravens are visual foragers, meaning that carcasses in open habitats are easy to locate from the sky and therefore are generally discovered before carrion in closed environments (Bragato et al. 2022; Higgins et al. 2006; Newsome and Spencer 2022). They are also well-known roadside scavengers and are regularly observed feeding on dead animals from vehicle collisions, particularly within Tasmania (Fielding et al. 2021a). In this study, ravens found and used almost all the carcasses within modified landscapes and only 80% of those in forested areas (Fig. 3b). Whilst ravens were more likely to discover carcasses on roadsides and farmland, likely due to their visual foraging style, there was no difference in carcass use or foraging time between habitats. This is likely due to reduced competition or perceived predation risk from larger scavengers and the increased persistence of carcasses across habitats. Previous work has found that the presence of top mammalian carnivores, particularly specialised scavengers such as the Tasmanian devil, can suppress forest raven carrion use by consuming carcasses before ravens discover them (Cunningham et al. 2018; Fielding et al. 2021b). Scavenging studies in the Simpson Desert observed a similar pattern to our study, with ravens detecting carcasses earlier in open habitats yet having little difference in carcass visitation time between open and closed habitats. The authors noted that this was potentially explained by low visitation from larger scavengers in both open and closed environments (Bragato et al. 2022). In the absence of native mammalian carnivores, as observed on the Bass Strait Islands, carrion is persisting for longer and therefore ravens could have more opportunities to use carcasses across all habitats.

Raptors were more likely to discover and use carcasses in farmland compared to forests or roads. This finding supports the foraging style of most raptors, which are visual hunters, locating prey and carrion whilst in flight (Potier et al. 2020). It also suggests that whilst land clearing may be detrimental to raptors due to habitat loss, decreased breeding success, and human persecution, a countervailing effect is that it might also provide better opportunities for scavenging (Dennis et al. 2011; Department of Primary Industries 2006; Peisley et al. 2017). However, culling of macropods with lead bullets and poison-based control of pest species with baits like anticoagulant rodenticides on farmland properties could have damaging flow-on effects for raptors (Lohr et al. 2020; Pay et al. 2021a; Woodford et al. 2020). For example, Pay et al. (2021b) found that all samples from 109 Tasmanian Wedge-tailed Eagle carcasses contained lead, with 10% of those samples having elevated concentrations of physiological concern. It is currently unclear if lead ingestion from hunter-killed macropods has any impact on raven populations in Australia, however, common raven (*Corvus corax*) populations in North America exhibited elevated blood lead levels during the hunting season in Yellowstone National Park (Craighead and Bedrosian 2008). Our study also found that raptors were more likely to scavenge on Flinders Island than on King Island, which is likely the result of a larger population of raptor species on Flinders Island (Fielding 2022).

Cats regularly use human-made tracks and roads for transportation and hunting (Dawson et al. 2018; Doherty et al. 2014). Whilst cats used more carcasses found on roadsides than those on farms or in forests within our study, there was no statistical difference in the discovery or usage rate of carcasses between habitats. Like forest ravens, this could be explained by reduced competition and increased persistence of carcasses on the islands. Previous work has found that cats will seek to avoid interspecific conflict with larger carnivores, such as the Tasmanian devil (Cunningham et al. 2018; Fancourt 2016; Lazenby and Dickman 2013). The lack of any habitat effect within our study system could suggest that in the absence of the top-down pressure exerted by native mammalian carnivores, cats do not prioritise modified habitats for hunting and scavenging. We also found that cats were more likely to use carcasses on Flinders Island than King Island which, like raptors, is possibly due to differences in population size of the two species, however, this has yet to be confirmed. Furthermore, the species is thought to rarely scavenge (Jones and Coman 1981; Paltridge et al. 1997). Our findings contradict those studies as we observed feral cats using almost half of all carcasses we deployed, which is also likely triggered by the lack of scavenging competition (Fielding et al. 2021b; Spencer et al. 2021). Does this mean that cats are prioritising scavenging over live wildlife in environments without native mammalian carnivores? Or does the presence of readily available carcasses simply supplement their population, further exacerbating impacts on small native fauna?

Despite carcasses within modified landscapes being used to a greater extent than those in forested environments, almost all carcasses across the three habitat types persisted until the end of the study period. Native mammalian carnivores are completely absent from the Bass Strait Islands following the human-driven eradication of the spotted-tailed quoll in the early twentieth century (Peacock et al. 2018). Previous work completed over equivalent periods (21 days) on mainland Tasmania found that carcasses within forested environments were quickly consumed when native mammalian carnivores were present, with most carcasses being completely consumed within three weeks (Cunningham et al. 2018; Fielding et al. 2021b). Our findings provide further support for the hypothesis that mesoscavengers are unable to replace the scavenging efficiency of these apex scavengers, despite their increased use of carcasses within modified landscapes. The more durable persistence of carcasses in landscapes with diminished or missing mammalian scavengers might also have adverse effects on human and animal health, due to the increased spread of carrion-borne diseases, particularly in agricultural areas (Moleón and Sánchez-Zapata 2021; Ogada et al. 2012).

Land-use change continues to shift the availability of carrion within landscapes. Here, we show how the discovery and use of carcasses differ between species and across habitats. Whilst avian scavengers appeared to discover more carcasses within modified landscapes, almost all carcasses persisted until the end of the study, likely due to the absence of specialised scavengers in the environment. This highlights the importance of apex mammalian scavengers within an environment and provides support for conservation strategies, such as trophic rewilding, and carrion management measures, such as carcass removal from paddocks and roadsides. In addition, the lack of any habitat effect on feral cats indicates that habitat restoration strategies might not be as effective in reducing the abundance of these synanthropic species as efforts to establish or bolster the presence of native mammalian carnivores. However, further research is needed to investigate the synergistic effects between habitat and the abundance of apex scavengers, to inform the most appropriate management strategies.

## Data availability statement

Data are available from the Zenodo Repository: 10.5281/zenodo.15130027.

## Supplementary Information

Below is the link to the electronic supplementary material.

Supplementary file1 (DOCX 216 KB)
