# Supplementary material for: Carcass use by mesoscavengers varied across modified landscapes in the absence of top carnivores
Source: Oecologia. 2025 Apr 3;207(4):60. doi: 10.1007/s00442-025-05697-1 (PMC11968553; doi:10.1007/s00442-025-05697-1)
Supplement: Supplementary file 1 — Supplementary file1 (DOCX 216 KB) [file 442_2025_5697_MOESM1_ESM.docx]

**Supplementary Information**

Carcass use by mesoscavengers varied across modified landscapes in the absence of top carnivores

*Matthew W. Fielding, Luke A. Yates, Jessie C. Buettel, Dejan Stojanovic and Barry W. Brook*

**Supplementary Table 1: Candidate model set for all analyses.** These model sets were used in Cox proportional hazards models against six different response variables – discovery of carcasses by all species; discovery of carcasses by forest ravens (*Corvus tasmanicus*); discovery of carcasses by feral cats (*Felis catus*); discovery of carcasses by raptors and persistence of carcasses. These model sets were also used in Generalised Linear Models against four different response variables – whether ravens fed at a carcass (GLM with a binomial distribution and a log link function); total scavenging time by ravens for the carcasses at which they fed (GLMs with a Gamma distribution and a log link function); whether feral cats fed at a carcass (GLM with binomial link function); and whether raptors fed at a carcass (GLM with binomial link function). All models included ‘island’ as a predictor to account for any variation between islands.

|  | **Model name** | **Model structure** |
| --- | --- | --- |
| 1 | null | island |
| 2 | p | pop + island |
| 3 | f | farm + island |
| 4 | r | road + island |
| 5 | w | dist.fw + island |
| 6 | c | dist.coast + island |
| 7 | fr | farm + road + island |
| 8 | fpr | farm + pop + road + island |
| 9 | fwr | farm + dist.fw + road + island |
| 10 | fcr | farm + dist.coast + road + island |
| 11 | pcw | pop + dist.coast + dist.fw + island |
| 12 | full | pop + farm + road + dist.fw + dist.coast + island |

**Supplementary Table 2: Carcass persistence and discovery model selection scores.** LOOCV (leave-one-out cross validation) results for Cox proportional hazards models assessing carcass persistence; carcass discovery by all species; carcass discovery by forest ravens; carcass discovery by raptors and carcass discovery by feral cats. Each section is ordered by complexity (degrees of freedom or number of estimated parameters) followed by model score. The selected model is in bold. model = model name/number; df = degrees of freedom; score = LOOCV-calculated model score estimate; del_score = difference between score of model and score of best scoring model; se_mod = correlation-adjusted standard error of the lowest estimated score.

|  | model | df | score | del_score | se_mod |
| --- | --- | --- | --- | --- | --- |
| *Carcass persistence* | | | | | |
|  | **null** | **1** | **52.48828** | **0** | **0** |
|  | c | 2 | 54.26912 | 1.780837 | 0.585556 |
|  | p | 2 | 55.16992 | 2.681633 | 1.960167 |
|  | w | 2 | 57.97389 | 5.485603 | 2.530403 |
|  | r | 2 | 85.82531 | 33.33703 | 29.01274 |
|  | f | 2 | 90.2054 | 37.71712 | 26.06414 |
|  | fr | 3 | 85.76973 | 33.28144 | 28.99085 |
|  | pcw | 4 | 63.58917 | 11.10089 | 3.86933 |
|  | fcr | 4 | 87.80907 | 35.32078 | 28.95421 |
|  | fpr | 4 | 91.41046 | 38.92218 | 31.33788 |
|  | fwr | 4 | 93.77625 | 41.28797 | 32.51297 |
|  | full | 6 | 119.4811 | 66.9928 | 47.73145 |
|  |  |  |  |  |  |
| *Carcass discovery* | | | | | |
| *All species* | | | | | |
|  | **null** | **1** | **1130.184** | **3.988973** | **4.729296** |
|  | r | 2 | 1127.805 | 1.610132 | 3.370433 |
|  | c | 2 | 1130.505 | 4.310197 | 4.771768 |
|  | p | 2 | 1131.269 | 5.074824 | 4.975512 |
|  | f | 2 | 1131.769 | 5.574381 | 4.243664 |
|  | w | 2 | 1132.157 | 5.962345 | 4.811873 |
|  | fr | 3 | 1126.195 | 0 | 0 |
|  | fpr | 4 | 1126.845 | 0.650801 | 1.586542 |
|  | fwr | 4 | 1127.524 | 1.32978 | 0.43863 |
|  | fcr | 4 | 1127.527 | 1.332291 | 2.305276 |
|  | pcw | 4 | 1133.242 | 7.047587 | 5.332071 |
|  | full | 6 | 1128.627 | 2.432325 | 2.743022 |
| *Forest ravens* | | | | | |
|  | null | 1 | 1086.062 | 12.80778 | 5.65835 |
|  | r | 2 | 1080.947 | 7.69299 | 4.399231 |
|  | f | 2 | 1085.372 | 12.11873 | 5.12206 |
|  | c | 2 | 1085.667 | 12.41299 | 5.660873 |
|  | w | 2 | 1088.302 | 15.04777 | 5.500961 |
|  | p | 2 | 1088.71 | 15.45609 | 5.620296 |
|  | **fr** | **3** | **1073.254** | **0** | **0** |
|  | fcr | 4 | 1074.115 | 0.861033 | 2.214973 |
|  | fwr | 4 | 1074.917 | 1.663472 | 0.346761 |
|  | fpr | 4 | 1075.828 | 2.574349 | 0.690097 |
|  | pcw | 4 | 1090.747 | 17.49366 | 5.54802 |
|  | full | 6 | 1078.109 | 4.855543 | 2.392777 |
| *Raptors* | | | | | |
|  | null | 1 | 247.8534 | 6.287455 | 4.227168 |
|  | **f** | **2** | **241.5659** | **0** | **0** |
|  | r | 2 | 246.8674 | 5.301455 | 3.524278 |
|  | w | 2 | 249.3135 | 7.747612 | 4.38082 |
|  | c | 2 | 249.8879 | 8.322025 | 4.30742 |
|  | p | 2 | 251.639 | 10.07314 | 4.070254 |
|  | fr | 3 | 243.7674 | 2.201451 | 0.709771 |
|  | fwr | 4 | 245.2959 | 3.730036 | 0.786543 |
|  | fcr | 4 | 245.9066 | 4.340742 | 0.977849 |
|  | fpr | 4 | 248.1897 | 6.623838 | 1.601547 |
|  | pcw | 4 | 255.1888 | 13.62289 | 4.214366 |
|  | full | 6 | 252.0251 | 10.45918 | 1.69467 |
| *Feral cats* | | | | | |
|  | **null** | **1** | **630.5917** | **2.39213** | **2.512531** |
|  | c | 2 | 628.1996 | 0 | 0 |
|  | r | 2 | 628.6688 | 0.469233 | 3.670513 |
|  | f | 2 | 632.0569 | 3.857324 | 2.692095 |
|  | w | 2 | 632.7301 | 4.530464 | 2.587889 |
|  | p | 2 | 633.3452 | 5.145598 | 2.980533 |
|  | fr | 3 | 630.7649 | 2.565299 | 3.747915 |
|  | fcr | 4 | 628.7269 | 0.527312 | 2.914036 |
|  | pcw | 4 | 632.4066 | 4.207058 | 2.264205 |
|  | fpr | 4 | 632.8172 | 4.617652 | 4.227624 |
|  | fwr | 4 | 633.5774 | 5.377798 | 4.03103 |
|  | full | 6 | 632.6168 | 4.417203 | 4.171368 |

**
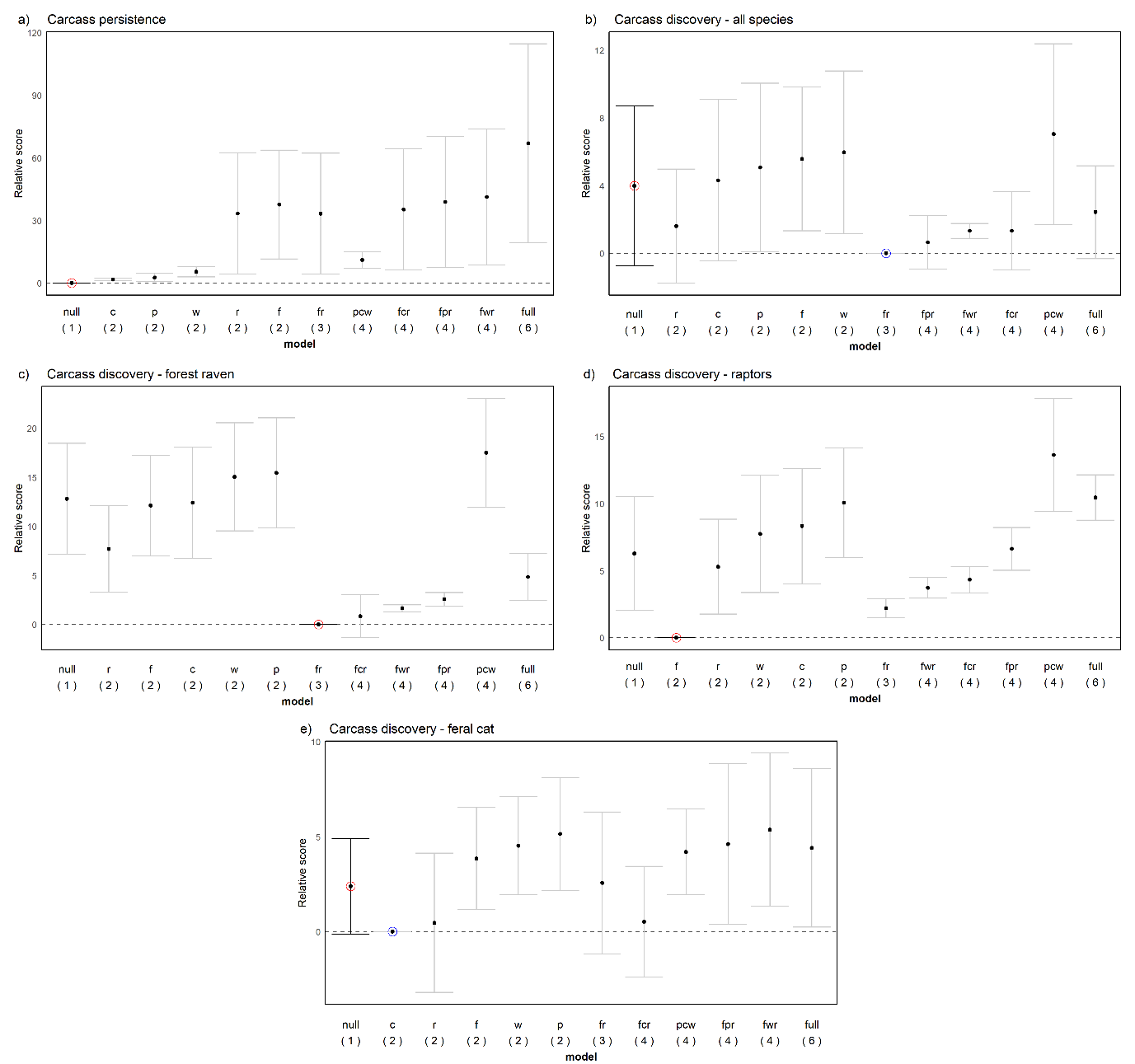
Supplementary Figure 1: Carcass persistence and discovery model selection scores.** Model score estimates with correlation-adjusted standard errors of Cox proportional hazards models for (a) carcass persistence; (b) carcass discovery by all species; (c) carcass discovery by forest ravens; (d) carcass discovery by raptors; and (e) carcass discovery by feral cats. Labels on the x-axis refer to model names ordered by increasing complexity (degrees as freedom as shown in brackets below). The blue circle indicates the model with the lowest score. The red circle indicates the model selected under the modified selection rule, which is the simplest model whose score estimate lies within one correlation-adjusted standard error of the lowest estimated score.

**Supplementary Table 3: Model results for carcass persistence and discovery.** Model output for the top selected model for carcass persistence and carcass discovery by all species, forest ravens, raptors, and feral cats. The table shows the df = degrees of freedom and model coefficient estimates ± standard error for each predictor.

| Preferred  model | df | Variable | Estimate |
| --- | --- | --- | --- |
| *Carcass persistence* | | | |
| null | 1 | island | -0.151 ± 1.002 |
|  |  |  |  |
| *Carcass discovery* | | | |
| *All species* | | | |
| null | 1 | island | 0.181 ± 0.187 |
| *Forest ravens* | | | |
| fr | 3 | farm | 0.787 ± 0.246 |
|  |  | road | 0.944 ± 0.245 |
|  |  | island | 0.139 ± 0.194 |
| *Raptors* | | | |
| f | 2 | farm | 1.241 ± 0.434 |
|  |  | island | 0.777 ± 0.458 |
| *Feral cats* | | | |
| null | 1 | island | 0.613 ± 0.270 |

**Supplementary Table 4: Carcass consumption and scavenging time model selection scores.** LOOCV (leave-one-out cross validation) results for generalised linear models assessing carcass use (binary) by feral cats, raptors, and forest ravens; and scavenging time (continuous) by forest ravens. Each section is ordered by complexity (degrees of freedom) followed by model score. The selected model is in bold. model = model name/number; df = effective degrees of freedom; score = LOOCV-calculated model score estimate; del_score = difference between score of model and score of best scoring model; se_mod = correlation-adjusted standard error of the lowest estimated score.

|  | model | df | score | del_score | se_mod |
| --- | --- | --- | --- | --- | --- |
| *Carcass use* | | | | | |
| *Feral cats* | | | | | |
|  | **null** | **2** | **136.9379** | **1.190177** | **2.43477** |
|  | c | 3 | 135.7477 | 0 | 0 |
|  | r | 3 | 135.8577 | 0.110038 | 4.125664 |
|  | w | 3 | 138.7217 | 2.974035 | 2.408782 |
|  | f | 3 | 138.8648 | 3.117126 | 2.595253 |
|  | p | 3 | 139.5996 | 3.851919 | 2.699931 |
|  | fr | 4 | 137.6846 | 1.936903 | 4.344297 |
|  | fcr | 5 | 136.9007 | 1.153025 | 3.153379 |
|  | fpr | 5 | 140.101 | 4.35333 | 4.446887 |
|  | fwr | 5 | 140.1914 | 4.443734 | 4.50087 |
|  | pcw | 5 | 140.2345 | 4.486763 | 1.283985 |
|  | full | 7 | 141.8298 | 6.082112 | 3.612016 |
| *Raptors* | | | | | |
|  | null | 2 | 94.88221 | 6.618542 | 4.542459 |
|  | **f** | **3** | **88.26367** | **0** | **0** |
|  | r | 3 | 91.70636 | 3.442689 | 4.530863 |
|  | c | 3 | 96.21814 | 7.95447 | 4.917167 |
|  | w | 3 | 96.66197 | 8.398297 | 4.815428 |
|  | p | 3 | 98.41591 | 10.15224 | 4.40498 |
|  | fr | 4 | 89.69863 | 1.434961 | 2.337166 |
|  | fcr | 5 | 91.10702 | 2.843356 | 2.642509 |
|  | fwr | 5 | 91.60909 | 3.345424 | 2.369939 |
|  | fpr | 5 | 94.47789 | 6.214218 | 2.428254 |
|  | pcw | 5 | 101.5221 | 13.25839 | 4.883566 |
|  | full | 7 | 98.02636 | 9.762688 | 2.561048 |
| *Forest ravens* | | | | | |
|  | **null** | **2** | **55.21294** | **0** | **0** |
|  | w | 3 | 56.13763 | 0.92468 | 0.81988 |
|  | c | 3 | 57.08751 | 1.874567 | 6.608393 |
|  | p | 3 | 57.31357 | 2.100629 | 1.040801 |
|  | r | 3 | 85.82583 | 30.61289 | 25.14096 |
|  | f | 3 | 86.20997 | 30.99702 | 25.08039 |
|  | fr | 4 | 115.0219 | 59.80891 | 33.59623 |
|  | pcw | 5 | 60.52053 | 5.307583 | 6.173692 |
|  | fwr | 5 | 117.9096 | 62.69664 | 34.6886 |
|  | fpr | 5 | 119.1438 | 63.93087 | 34.54545 |
|  | fcr | 5 | 120.0506 | 64.83766 | 39.87754 |
|  | full | 7 | 123.4829 | 68.26992 | 39.21796 |
|  | | | | | |
| *Scavenging time* | | | | | |
| *Forest ravens* | | | | | |
|  | **null** | **3** | **1433.712** | **1.134111** | **1.320368** |
|  | w | 4 | 1432.578 | 0 | 0 |
|  | c | 4 | 1434.864 | 2.286125 | 1.506282 |
|  | r | 4 | 1435.182 | 2.603912 | 1.347209 |
|  | f | 4 | 1435.229 | 2.650965 | 1.38751 |
|  | p | 4 | 1436.312 | 3.734185 | 2.136453 |
|  | fr | 5 | 1436.939 | 4.360739 | 1.419902 |
|  | fwr | 6 | 1435.813 | 3.23479 | 0.342797 |
|  | pcw | 6 | 1435.943 | 3.364946 | 1.403063 |
|  | fcr | 6 | 1438.197 | 5.61956 | 1.627067 |
|  | fpr | 6 | 1439.805 | 7.227061 | 2.311102 |
|  | full | 8 | 1439.496 | 6.918298 | 1.599081 |

**
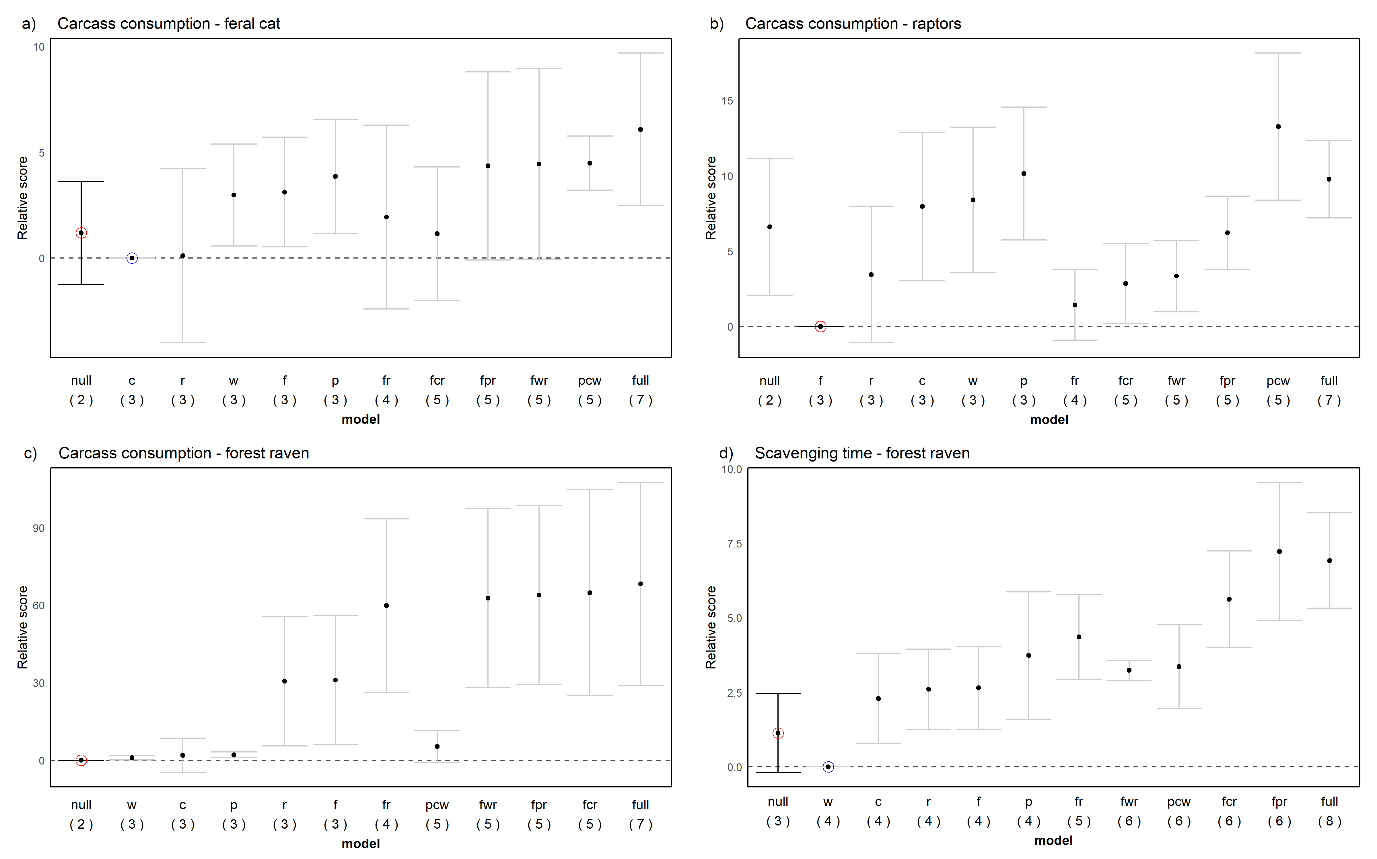
**

**Supplementary Figure 2: Carcass consumption and scavenging time model selection scores.** Model score estimates with correlation-adjusted standard errors for generalised linear models assessing carcass use by (a) feral cats; (b) raptors; and (c) forest ravens; and (d) scavenging time by forest ravens. Labels on the x-axis refer to model names ordered by increasing complexity (degrees of freedom as shown in brackets below). The blue circle indicates the model with the lowest score. The red circle indicates the model selected under the modified selection rule, which is the simplest model whose score estimate lies within one correlation-adjusted standard error of the lowest estimated score.

**Supplementary Table 5: Model results for carcass consumption and scavenging time.** Model output for the top selected model for carcass use by feral cats, raptors and forest ravens and scavenging time for forest ravens. The table shows the df = degrees of freedom and model coefficient estimates ± standard error for each predictor.

| Preferred  model | df | Variable | Estimate |
| --- | --- | --- | --- |
| *Carcass consumption* | | | |
| *Feral Cats* | | | |
| null | 2 | Intercept | -0.511 ± 0.298 |
|  |  | island | 0.981 ± 0.412 |
| *Raptors* | | | |
| f | 3 | Intercept | -2.944 ± 0.608 |
|  |  | farm | 1.635 ± 0.568 |
|  |  | island | 1.210 ± 0.604 |
| *Forest ravens* | | | |
| null | 2 | Intercept | 2.398 ± 0.522 |
|  |  | island | 0.395 ± 0.791 |
|  | | | |
| *Scavenging time* | | | |
| *Forest ravens* | | | |
| w | 3 | Intercept | 6.828 ± 0.118 |
|  |  | island | -0.269 ± 0.163 |
